# The impact of COVID-19 on the mental health of older sexual minority Canadians in the CLSA

**DOI:** 10.1186/s12877-023-04513-w

**Published:** 2023-12-07

**Authors:** Alexandra Grady, Arne Stinchcombe

**Affiliations:** 1 School of Psychology, University of Ottawa, Ottawa, ON Canada; 2 Bruyère Research Institute (BRI), Ottawa, ON Canada

**Keywords:** LGB, Aging, Depression, Loneliness, COVID-19 pandemic, CLSA

## Abstract

**Purpose:**

Limited research has examined the mental health impacts of the COVID-19 pandemic on sexual minority (i.e., lesbian, gay, bisexual; LGB) older adults using a longitudinal approach. This study aimed to compare the mental health trajectories (i.e., reported symptoms of depression and loneliness) of LGB and heterosexual participants across four time points.

**Methods:**

Data were drawn from the Canadian Longitudinal Study on Aging (CLSA), an ongoing study on the experiences of adults between 45 to 85 at Baseline. Data included for analysis were collected at Baseline (2011-2015), Follow-up 1 (2015-2018), and two time points during the COVID-19 pandemic (April-December 2020). We used General Estimating Equations (GEE) to model changes in depression symptoms (CESD-10; *n* = 47,728) and loneliness (UCLA 3-item loneliness scale; *n* = 41,698), adjusting for covariates (i.e., age, sex, race/ethnicity, education, and income).

**Results:**

Results indicated that LGB participants reported more symptoms of depression (*B* = .595, *p* < .001) and loneliness (*B* = .313, *p* < .001) in comparison to heterosexual peers across the four time points. Mean depression and loneliness scores increased regardless of sexual orientation.

**Conclusion:**

This study highlighted the detrimental effects of the pandemic on the mental health of older adults regardless of sexual orientation. It also showed that LGB older adults experienced more loneliness and depression symptoms than heterosexual older adults both before and during the pandemic. Understanding diverse identities, needs, and disparities in mental health is critical to promoting equitable aging experiences for everyone.

## Introduction

In addition to mortality and morbidity caused by COVID-19 infections, the pandemic has also resulted in poor mental health outcomes worldwide. In a systematic review, Vindegaard and Benros (2020) found greater levels of depression and anxiety in the general population during the pandemic compared to pre-pandemic periods [1]. One meta-analysis estimated the prevalence of depression and anxiety to be 33.75% and 31.9%, respectively [2]. These findings hold true for older adults. A survey of older adults in San Francisco found that 54% experienced worsened loneliness during the pandemic, which was associated with greater depression and anxiety symptoms [3]. A large longitudinal study in England also found that reported depression symptoms and loneliness, measured using a version of the Center for Epidemiological Studies Depression (CESD) scale and the UCLA loneliness scale, increased substantially in older adults during the pandemic [4]. A survey of older Canadians during the pandemic found moderate or clinically high depressive symptoms in over 43% of participants, with greater loneliness predicting worse depression trajectories [5].

In Canada, the COVID-19 pandemic spurred restrictions on travel, businesses, and gatherings. Between March and May 2020, most provinces and territories had closed businesses deemed “non-essential” (e.g., restaurants, retail stores) and restricted the number of people allowed at social gatherings [6]. In some parts of Canada, social gatherings of all sizes were prohibited while other areas permitted small gatherings. Recreation centres and worship services were also limited during this time. Between September and December 2020, provincial and territorial restrictions continued with many provinces implementing a region-based approach, allowing gatherings and businesses to open based on local infection rates [6]. This meant that people living in larger urban centres with higher rates of infection (e.g., Toronto) were often subject to tighter restrictions in comparison to people residing in smaller towns. In December 2020, a second wave of COVID-19 led provinces and territories to re-instate strict gathering limits and business closures that had gradually loosened up until that point. While everyone was affected by the pandemic, it had a greater negative impact on particular groups.

There is evidence that sexual minorities (i.e., people who identify as lesbian, gay, or bisexual; LGB) experienced worse mental health outcomes during the pandemic compared to non-LGB people. For example, a study of heterosexual and LGB adults aged 18 to 74 in Portugal and Brazil found that LGB participants reported more depression symptoms than heterosexual participants [7]. A survey in the U.S. during the first wave of the pandemic found that LGB participants reported significantly more symptoms of depression and anxiety than heterosexual participants; in comparison to heterosexual participants, a significantly higher proportion of LGB participants’ scores met or exceeded the threshold for clinical concern [8]. Further, a large study of heterosexual and LGB adults in the U.S. found that sexual minority men and bisexual women reported poorer mental health both before and during the pandemic [9]. An online survey during the first two waves of the COVID-19 pandemic found that LGB people reported more loneliness and depression symptoms than their heterosexual counterparts [10]. These mental health disparities have also been found in Canada; a repeated cross-sectional monitoring survey of Canadians during the pandemic found that a greater proportion of LGB respondents reported a deterioration in mental health than heterosexual respondents [11, 12].

LGB older adults face unique challenges that can impact mental well-being. In research conducted pre-pandemic, LGB older adults in Canada reported more depression symptoms on the CESD compared to heterosexual peers [13]. They were also more likely to report having a mood or anxiety disorder [14]. Literature on the mental health of older LGB people during the COVID-19 pandemic continues to grow. For example, LGB older adults in the Health and Retirement Study in the United States reported more emotional stress, less in-person social contact, and more loneliness than heterosexual older adults [15]. A study of older LGB women in the UK found that 54% reported worsened mental health during mandatory social isolation [16]. A recent systematic review of the mental and physical health of older LGB adults during the pandemic found negative impacts to psychological health in the majority of research reviewed [17].

These mental health disparities stem from minority stress experiences associated with LGB identities. Minority stress theory holds that individuals who identify as LGB face stigma, discrimination, and other stressors that negatively impact health [18, 19]. These chronic, socially-based stressors accumulate across the lifespan and result in poorer health outcomes for LGB people [18, 20]. LGB older adults have previously been found to have a higher prevalence of living alone than heterosexual older adults [21]; living alone has been associated with loneliness in LGB older adults [22]. Among older adults in general, there is a strong association between UCLA loneliness scores and CESD-measured symptoms of depression [23]. The restrictions imposed during the COVID-19 pandemic, including the closure of programs and services for social support (e.g., senior centres), may have exacerbated feelings of loneliness and symptoms of depression in LGB older adults.

Understanding the impact of the COVID-19 pandemic on older LGB people in Canada is a critical step in responding to the needs of a diverse aging population. The present study aimed to contribute to our knowledge on the mental well-being of LGB older adults during the pandemic. Specifically, we asked two questions: (1) How did depression scores change over the course of the pandemic, and was there a disparity among LGB people? and (2) How did loneliness scores change over the course of the pandemic, and was there a disparity among LGB people? It was hypothesized that: (1) depression and loneliness would increase from before the pandemic (i.e., Baseline/Follow-up 1) to during the pandemic (i.e., COVID Baseline/Exit) across all participants, regardless of sexual orientation; (2) given minority stress experiences [18, 19], LGB participants will report greater depression and loneliness over time compared to heterosexual participants; and (3) given predisposing factors (e.g., living alone) and restrictions to in-person LGB-inclusive programming in combination with minority stress experiences, there will be a significant difference between the depression and loneliness trajectories of older LGB people in comparison to heterosexual people, such that LGB participants’ slope will be steeper from before the pandemic to during the pandemic (i.e., an interaction effect).

## Methods

### Data source

Data were drawn from the Canadian Longitudinal Study on Aging (CLSA), a study documenting age-related changes of approximately 50,000 adults aged 45 to 85 at Baseline every three years for 20 years. The sampling frame and strategy has been described in-depth elsewhere [24]. Baseline data were collected between 2011–2015, and Follow-up 1 data were collected between 2014–2018. At the outset of the pandemic, all CLSA participants were contacted by email to take part in the COVID-19 data collection cycle; COVID-19 Baseline data were collected between April 15^th^ and May 20^th^, 2020, and COVID-19 Exit data were collected between September 29^th^ and December 29^th^, 2020. The term “Exit” in this context denotes the final wave of COVID-specific surveys, not an exit interview. The COVID-19 surveys were administered through online questionnaires or telephone interviews based on participant preference. Written informed consent was obtained from each participant prior to data collection. The CLSA received ethics approval by research ethics boards (REBs) at each of the national collection sites, and the analyses described here were approved by the University of Ottawa’s REB (H-04–22-8051).

### Measures

#### Outcomes

##### Depressive symptoms

Depression symptoms were measured using the Center for Epidemiologic Studies Depression Scale short-form (CESD-10) [25]. The scale consists of ten statements, each ranked on a scale from zero (“rarely or none of the time”) to three (“all of the time”). The statements are summed to provide a total score ranging from zero to 30; higher scores indicate greater severity of depression, with a value of ten or greater indicating significant depressive symptoms [25]. The CESD-10 is considered a reliable and valid measure of depression symptoms in community-dwelling older adults (α = 0.70) [26]. The CESD-10 was administered during each of the four time points.

##### Loneliness

Loneliness was measured using the UCLA 3-item Loneliness Scale. This scale consists of three questions, each rated on a three-point scale from one (“hardly ever”) to three (“often”). Items are summed to give a total score ranging from three to nine; higher scores indicate greater loneliness. The UCLA 3-item Loneliness Scale is considered a reliable and valid measure of loneliness in community-dwelling older adults (α = 0.72) [27]. The UCLA 3-item Loneliness Scale was administered at two time points, Follow-up 1 and COVID-19 Exit.

### Explanatory variables

Given the research questions, sexual orientation was treated as a primary predictor. In the selection of covariates we drew from the Commission on Social Determinants of Health (CSDH) conceptual diagram which highlights the role of socioeconomic position (e.g., gender, race/ethnicity, education, income) on health outcomes [28].

#### Sexual orientation

Sexual orientation was the focal variable of interest. Sexual orientation was ascertained by asking the question: “Do you consider yourself to be: Heterosexual? Homosexual? Bisexual?”. In order to maximize cell sizes representing LGB participants, participants who identified as homosexual or bisexual were grouped together to form an “LGB” variable. Participants were included if they reported their sexual orientation at Baseline or Follow-up 1; participants who refused to answer or who responded with “don’t know” or “none of the above” were excluded from the analysis (*n* = 76).

#### Covariates

Covariates included age (years) at Baseline, sex, income, education, and race/ethnicity. Sex was determined at Baseline by asking the question: “Are you male or female?”. Income was determined by asking, “What is your best estimate of the total household income received by all household members, from all sources, before taxes and deductions, in the past 12 months?” We treated income as a categorical variable with five levels (in Canadian dollars): < $20,000, $20,000–49,999, $50,000–99,999, $100,000–149,999, and >  = $150,000. Participants’ highest level of education was grouped into four categories: “less than secondary school graduate”, “secondary school graduate”, “some post-secondary” and “post-secondary graduate”. In Canada, secondary school is equivalent to high school in other regions, and post-secondary school is equivalent to college or university. Race/ethnicity was collected by asking “People living in Canada come from many different cultural and racial backgrounds. Are you…[list of cultural/racial backgrounds]?” We classified participants as either “white” or “non-white” to maintain adequate cell sizes.

### Statistical analysis

All statistical analyses were conducted in Stata/SE Version 15.1 [29]. Means and standard deviations of basic demographic variables are listed in Table 1. To determine differences between heterosexual and LGB groups, independent *t*-tests were used for continuous variables and chi-square tests (χ^2^) were computed for categorical variables.

**Table 1:** Demographic and mental health variables by sexual orientation

	**Total** *n* = 47,728^a^ *n *(%) or *M* (SD)	**Heterosexual** *n* = 46,709^a^* n *(%) or *M* (SD)	**LGB** *n* = 1,019^a^ *n *(%) or *M* (SD)	**Test statistic and ***p*-value
**Characteristics**				
***Demographic variables***				
Age at Baseline (Range: 45–85)	62.7 (10.4)	62.8 (10.4)	59.3 (9.1)	*t* = 10.64, *p* < .001
Sex				χ^2^ = 65.40, *p* < .001
Male	23,855 (50.0)	23,218 (49.7)	637 (62.5)	
Female	23,873 (50.0)	23,491 (50.3)	382 (37.5)	
Income				χ^2^ = 17.04, *p* = .002
< $20 K	2,854 (6.0)	2,764 (5.9)	90 (8.8)	
$20–49,999	12,085 (25.3)	11,832 (25.3)	253 (24.8)	
$50–99,999	17,046 (35.7)	16,686 (35.7)	360 (35.3)	
$100–149,999	8,716 (18.3)	8,530 (18.3)	186 (18.3)	
≥ $150 K	7,027 (14.7)	6,897 (14.8)	130 (12.8)	
Education				χ^2^ = 29.57, *p* < .001
< Secondary school	3,292 (6.9)	3,258 (7.0)	34 (3.3)	
Secondary school	5,249 (11.0)	5,162 (11.1)	87 (8.5)	
Some post-secondary	3,593 (7.5)	3,508 (7.5)	85 (8.3)	
Post-secondary	35,594 (74.6)	34,781 (74.5)	813 (79.8)	
Race				χ^2^ = 0.13, *p* = .72
Non-white	2,314 (4.8)	2,267 (4.9)	47 (4.6)	
White	45,414 (95.2)	44,442 (95.1)	927 (95.4)	
***Mental health variables***				
Depression symptoms				
Baseline	5.3 (4.7)	5.3 (4.6)	6.0 (5.3)	*t* = -4.63, *p* < .001
Follow-up 1	5.1 (4.6)	5.1 (4.6)	6.0 (5.1)	*t* = -5.60, p < .001
COVID-19 Baseline	6.0 (5.2)	6.0 (5.1)	7.0 (5.9)	*t* = -4.95, *p* < .001
COVID-19 Exit	6.1 (5.2)	6.1 (5.2)	6.9 (6.0)	*t* = -3.64, *p* < .001
Loneliness symptoms				
Follow-up 1	3.8 (1.3)	3.8 (1.3)	4.2 (1.5)	*t* = -7.73, *p* < .001
COVID-19 Exit	4.3 (1.6)	4.3 (1.5)	4.6 (1.7)	*t* = -4.36, *p* < .001

*Note.* ^a^Depression analytic sample reported. Analytic sample for loneliness is 41,698

We used Generalized Estimating Equations (GEE) for the main analyses using the xtgee command in Stata. GEE allowed observations to be grouped by individual and by LGB status. We generated separate GEEs for depression symptoms and loneliness, with sexual orientation considered a primary predictor. Given the possibility that the relationship between sexual orientation and mental health depended on time point, a sexual orientation × time interaction was entered. The analytic sample for the model treating depression symptoms as the outcome was *n* = 47,728. The analytic sample for the model treating loneliness as the outcome was *n* = 41,698. We handled missing data through listwise deletion. Alpha was set to 0.05.

## Results

### Study population characteristics

Sample characteristics are shown in Table 1. The sample was largely white (*n* = 45,414; 95.2%) and reported completing post-secondary education (*n* = 35,594; 74.6%). Approximately 2% of participants identified as LGB (*n* = 1,019). LGB participants were younger (*M* = 59.3) than heterosexual participants (*M* = 62.8;* p* < 0.001). LGB participants reported higher levels of education in comparison to heterosexual participants (79.8% of LGB participants completed post-secondary school education; 74.5% of heterosexual participants completed post-secondary school; *p* < 0.001). More LGB participants were male (*n* = 637; 62.5%) than female (*n* = 382; 37.5%).

### Depression symptoms

**Fig. 1:**
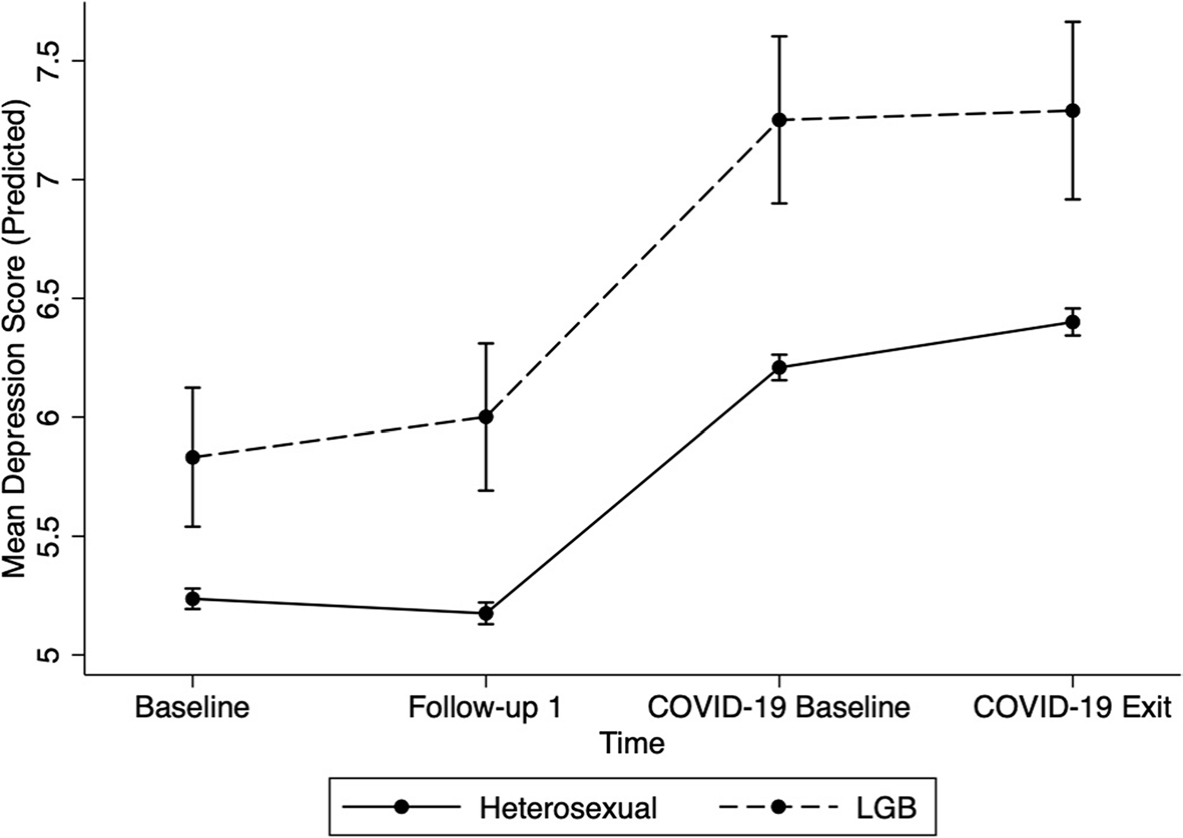
Predicted depression scores at each time point by sexual orientation

The model treating depression symptoms as the outcome reached statistical significance (Wald χ^2^ = 6112.41, *p* < 0.001). The results are summarized in Table 2. Results showed that LGB participants reported more symptoms of depression overall in comparison to heterosexual participants, after controlling for age, sex, race/ethnicity, income, and education (*B* = 0*.*595, *p* < 0.001). In addition, female participants reported more symptoms of depression than male participants (*B* = 0.8, *p* < 0.001). Participants with an income over $150,000 reported less symptoms of depression in comparison to those with an income under $20,000 (*B* = -3.7, *p* < 0.001). White participants reported fewer symptoms of depression than non-white participants (*B* = -0.2, *p* = 0.006). Overall, participants reported more depression symptoms both at COVID-19 Baseline (*B* = 1.0, *p* < 0.001) and COVID-19 Exit (*B* = 1.2, *p* < 0.001) in comparison to the first Baseline time point. The overall interaction between time and LGB status was not statistically significant (χ^2^ = 6.58, *p* = 0.086). Figure 1 shows the predicted values of depression symptoms over the four time points by sexual orientation.

**Table 2:** GEE models of demographic and time variables in relation to mental health measures

**Characteristics**	**Depression (*n* = 47,728)**			**Loneliness (*n* = 41,698)**		
	*B (SE)*	CI	*p*	*B (SE)*	CI	*p*
***Demographic variables***						
Age	-0.03 (0.002)	(-.04, -.03)	< .001	-0.008 (0.001)	(-0.009, -0.006)	< .001
Sex						
Male	Referent	-	-	Referent	-	-
Female	0.8 (.04)	(0.8, 0.9)	< .001	0.2 (0.01)	(0.1, 0.2)	< .001
Sexual orientation						
Heterosexual	Referent	-	-	Referent	-	-
LGB	0.6 (0.2)	(0.3, 0.9)	< .001	0.3 (0.05)	(0.2, 0.4)	< .001
Income						
< $20 K	Referent	-	-	Referent	-	-
$20–49,999	-1.8 (0.09)	(-1.9, -1.6)	< .001	-0.5 (0.03)	(-0.6, -0.5)	< .001
$50–99,999	-2.7 (0.08)	(-2.9, -2.6)	< .001	-0.8 (0.03)	(-0.9, -0.8)	< .001
$100–149,999	-3.2 (0.09)	(-3.4, -3.1)	< .001	-1.03 (0.03)	(-1.2, -1.0)	< .001
≥ $150 K	-3.7 (0.09)	(-3.8, -3.5)	< .001	-1.1 (0.03)	(-1.2, -1.1)	< .001
Education						
< Secondary school	Referent	-	-	Referent	-	-
Secondary school	-0.4 (0.09)	(-0.6, -0.3)	< .001	-0.1 (0.03)	(-0.2, -0.03)	.003
Some post-secondary	-0.2 (0.1)	(-0.3, -0.04)	0.124	0.02 (0.04)	(-0.05, 0.09)	.575
Post-secondary	-0.5 (0.08)	(-0.6, -0.3)	< .001	0.004 (0.03)	(-0.05, 0.06)	.876
Race						
Non-white	Referent	-	-	Referent	-	-
White	-0.2 (0.09)	(-0.4, -0.07)	.006	-0.1 (0.03)	(-0.2. -0.05)	< .001
***Time period***						
Baseline	Referent	-	-	-	-	-
Follow-up 1	-0.06 (0.02)	(-0.1, -0.02)	.008	Referent	-	-
COVID-19 Baseline	1.0 (0.03)	(0.9, 1.0)	< .001	-	-	-
COVID-19 Exit	1.2 (0.03)	(1.1, 1.2)	< .001	0.5 (0.009)	(0.5, 0.5)	< .001
***Interaction***						
Time x LGB (Overall)	χ^2^ = 6.58, p = .086			χ^2^ = 0.48, p = .490		
**Model Fit**	χ^2^ = 6112.41, *p* < .001			χ^2^ = 4983.02, *p* < .001

### Loneliness

**Fig. 2:**
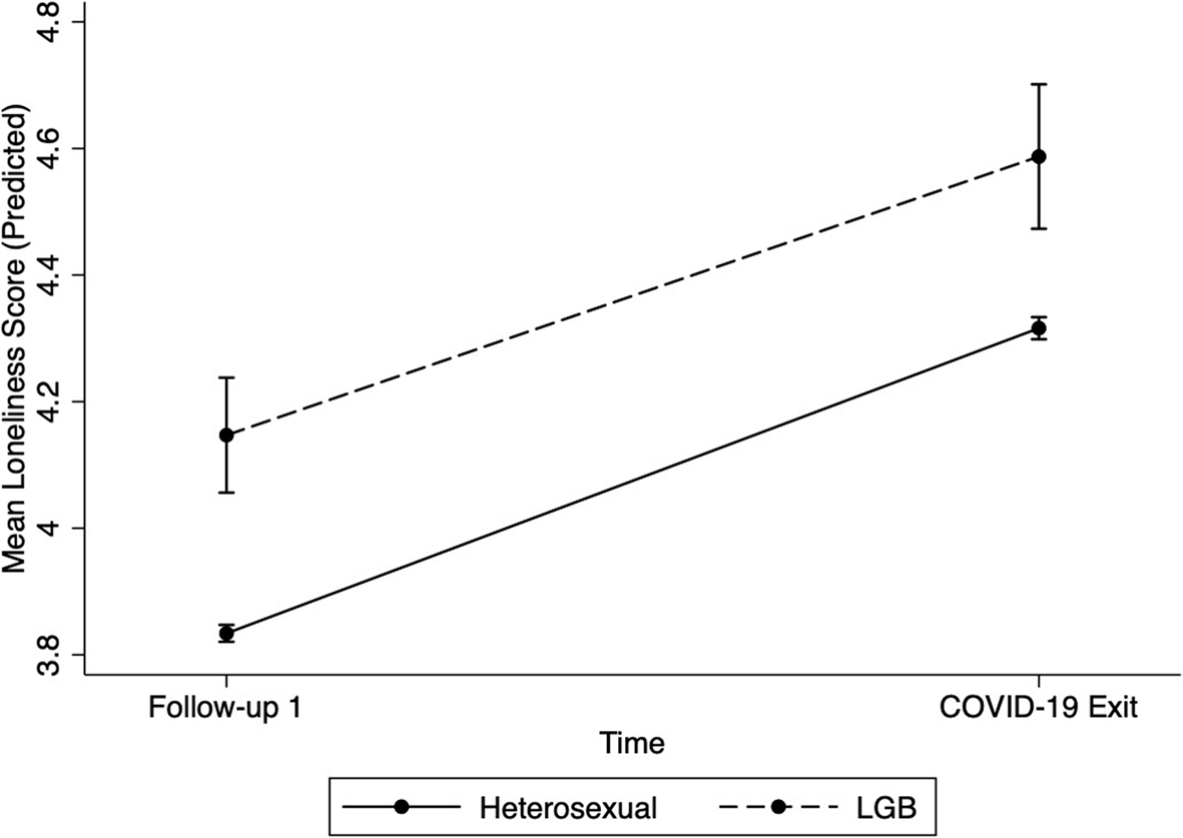
Predicted loneliness scores at each time point by sexual orientation

The model treating loneliness as the outcome reached statistical significance (Wald χ^2^ = 4983.02, *p* < 0.001). The results are summarized in Table 2. The results showed that LGB participants reported more loneliness overall in comparison to heterosexual participants, after controlling for age, sex, race/ethnicity, income, and education (*B* = 0.3, *p* < 0.001). Female participants reported more loneliness than male participants (*B* = 0.2, *p* < 0.001). Higher income was associated with less loneliness; the greatest difference was between participants with an income over $150,000 in comparison to participants with an income under $20,000 (*B* = -1.1, *p* < 0.001). White participants reported less loneliness than non-white participants (*B* = -0.1, *p* < 0.001). Overall, participants reported more loneliness at COVID-19 Exit than Follow-up 1 (*B* = 0.5, *p* < 0.001). The overall interaction between time and LGB status was not statistically significant (χ^2^ = 0.48, *p* = 0.490). Figure 2 shows the predicted values of loneliness symptoms over the two time points by sexual orientation.

## Discussion

In addition to the effects of the virus itself, the COVID-19 pandemic had impacts on mental health. The purpose of this study was to examine the mental health trajectories of older LGB and heterosexual Canadians during the COVID-19 pandemic. We hypothesized that, given the potential for minority stress experiences and their impact on health outcomes,^18,19^ LGB older people would fare poorer (i.e., have higher depression and loneliness scores) than heterosexual participants over the course of the pandemic. Our study yielded several noteworthy findings.

First, consistent with our hypothesis, LGB participants reported more depression symptoms and loneliness across all time points in comparison to heterosexual participants. This aligns with previous work on LGB older adults' mental health [13, 14] and the mental health of LGB people during the pandemic [7, 16]. Specifically, these results corroborate Fish et al.’s (2021) findings that LGB adults in the U.S. experienced poorer mental health both prior to and during the pandemic [9]. They are also in line with Westwood et al.’s (2021) finding that the majority of older lesbian and gay women reported worsening mental health during shelter-in-place orders in the UK [16].

These disparities stem from the lifetime accumulation of stigma, discrimination, and stressors that are unique to LGB people (i.e., minority stress experiences) [18, 19]. In the Minority Stress Model, Meyer (2003) postulates that distal (e.g., discrimination, violence) and proximal processes (e.g., identity concealment) interact to impact the mental health of LGB people [18]. In this case, the accumulation of stigma and discrimination across the lifespan resulted in higher scores on measures of depression symptoms and loneliness in older LGB Canadians in comparison to heterosexual Canadians across the four time points.

Second, while depression and loneliness scores were higher for LGB participants than heterosexual participants, there was an overall trend of worsening mental health, regardless of sexual orientation. Overall, participants reported greater depression symptoms and loneliness during the pandemic compared to pre-pandemic periods. This echoes other research findings of the declining mental health of the general population [1, 2], LGB people [8, 9], and older adults [4, 5, 30, 31] during the pandemic.

Third, the interaction between sexual orientation and time was not statistically significant, suggesting that the LGB participants’ mental health trajectory did not differ significantly from heterosexual participants. It is possible that aspects of the pandemic, such as mandatory shelter-in-place orders, may have limited experiences of discrimination typically encountered outside of the home. Qualitative work has described how some older LGB women felt that their mental health stayed the same or improved during the pandemic due to adaptation to new technologies (e.g., connecting with friends over video call instead of in person) and more time alone [16]. This highlights the adaptability and resiliency of LGB communities.

Additionally, our findings suggest differential mental health trajectories based on social determinants beyond sexual orientation. Younger, lower income, non-white, and female participants reported more depression symptoms and loneliness than older, higher income, white, and male participants. Other work has highlighted the role of social determinants of health as predictors of poorer outcomes during the pandemic. For example, in their evidence brief on the role of social determinants on health equity during the pandemic, the World Health Organization (WHO) highlighted the unequal burden of COVID-19 infection and death on poorer populations, disadvantaged ethnic groups, and older people, among others [32].

This study had a number of strengths, including a large population-based sample, multiple time points before and during the pandemic, and the use of validated measures of mental health (i.e., CESD-10 and UCLA 3-Item Loneliness Scale). The time points of the CLSA’s COVID-19 surveys overlapped with enhanced public health restrictions in Canada. Even as restrictions were lifting (during the COVID-Exit survey), depression scores remained elevated. The loneliness scores reported at this time may reflect a hesitation to gather with family and friends with a second wave of COVID-19 looming. The timing of these surveys provided insight into the experiences of older Canadians under those circumstances.

The present study also carried several limitations. The majority of participants reported being white, completing post-secondary education, and earning a moderate-to-high income. Despite a large sample, the small proportion of LGB people precluded separate analyses of lesbian, gay, and bisexual groups, potentially ignoring the diverse experiences of each group. Our analysis was further limited by the CLSA’s question on sexual orientation. While sexuality is multidimensional (e.g., encompassing identity, attraction, and behaviour [33]), the CLSA’s question centred around sexual identity. Participants may have reported a heterosexual identity, which may not exactly capture their sexual attraction and/or behaviour. Though the literature suggests that minority stress experiences underly the discrepancy in depression and loneliness scores in LGB participants, these experiences were not directly captured in CLSA data collection and therefore sexual orientation was used as a proxy measure of minority stress. While this study was focused on reporting disparities in mental health throughout the pandemic, there is a robust and growing body of work highlighting the strengths exhibited by members of these communities, including strong social networks and adaptability [16, 34, 35]. Future work should take a strengths-based approach, examining the resilience exhibited by members of these communities in times of crisis.

## Conclusion

Older adults were disproportionately impacted by COVID-19 infections and mortalities. This study highlighted the detrimental effects of the pandemic on the mental health of older adults regardless of sexual orientation. It also showed that LGB older adults experienced more loneliness and depression symptoms than heterosexual older adults both before and during the pandemic. Understanding diverse identities, needs, and disparities in mental health is critical to promoting equitable aging experiences for everyone. Further research, development, and testing of interventions aimed at improving mental health in older LGB people is needed.

## Data Availability

The data and materials are available to approved data users. More information about data access can be found at the following link: https://www.clsa-elcv.ca/data-access.
